# Maine Medical and Surgical Reporter

**Published:** 1858-09

**Authors:** 


### MAINE MEDICAL AND SURGICAL REPORTER.

Having been unable to give much attention to our Journal of late, we had failed to notice this new candidate for the favor of the profession. We have received the first and second numbers, commencing with June, and we can recommend it as an exceedingly promising addition to the list of Medical Journals of the country. An interesting report of the proceedings of the Maine Medical Association, taken from its pages, will be found under the head of selections.

It contains 48 octavo pages and will be published monthly at Portland, Maine, at three dollars a year payable in advance. Address the editor.

